# The Silent Strangulation: Post-hysterectomy Ureteral Endometriosis Masquerading as Iatrogenic Injury

**DOI:** 10.7759/cureus.104421

**Published:** 2026-02-27

**Authors:** Prabhkaran Singh Gill, Pareena Kaur Gill, Ishwinder S Hanjra, Aditya Partap Singh, Parmeesh Kaur Bansal, Shubhkaran Singh Gill

**Affiliations:** 1 Internal Medicine, Adesh Institute of Medical Sciences and Research, Bathinda, IND; 2 Medicine, Adesh Institute of Medical Sciences and Research, Bathinda, IND; 3 Medicine, Dayanand Medical College and Hospital, Ludhiana, IND; 4 Internal Medicine, Sri Guru Ram Das Institute of Medical Sciences and Research, Amritsar, IND; 5 General Surgery, Dayanand Medical College and Hospital, Ludhiana, IND

**Keywords:** deep infiltrating endometriosis, diagnostic delay, hydroureteronephrosis, iatrogenic ureteral injury, obstructive uropathy, post-hysterectomy, rare case report, ureteral endometriosis, ureteral obstruction, uterine adenomyosis

## Abstract

Ureteral endometriosis (UE) is a rare but potentially devastating manifestation of deep infiltrating endometriosis, often remaining asymptomatic until irreversible renal damage occurs. We present the case of a 42-year-old woman with a long-standing history of obstetric loss and severe menorrhagia. She underwent a total abdominal hysterectomy (TAH) for extensive adenomyosis with multifocal pelvic endometriosis, confirmed on histopathology. While her uterine symptoms resolved postoperatively, she returned three months later with persistent right-sided flank and groin pain. Given her recent surgery, initial clinical suspicion centered on iatrogenic ureteral injury or recurrent urinary tract infection, leading to repeated courses of antibiotics with no relief. Advanced imaging ultimately revealed Grade IV hydroureteronephrosis with characteristic distal ureteral tapering, confirming mechanical obstruction. Importantly, the obstruction was not surgical in origin; it reflected the progression of deep infiltrating endometriosis involving the ureteral adventitia, likely present before the hysterectomy. Postoperative fibrosis may have exacerbated the ureteral constriction, precipitating the acute presentation. This case highlights a critical diagnostic pitfall: UE can remain clinically silent until post-surgical changes unmask obstruction, mimicking more common postoperative complications. We advocate for proactive preoperative evaluation of the ureters in patients with extensive endometriosis to prevent silent loss of renal function. Even after definitive uterine surgery, clinicians must maintain a high index of suspicion for ureteral involvement. Early recognition and intervention are essential to prevent irreversible kidney injury.

## Introduction

Endometriosis is a chronic, estrogen-dependent inflammatory condition characterized by the presence of functional endometrial glands and stroma outside the uterine cavity. It affects approximately 6-10% of women of reproductive age and up to 50% of women with chronic pelvic pain or infertility [1]. Deep infiltrating endometriosis is the most aggressive phenotype, defined as lesions penetrating more than 5 mm beneath the peritoneal surface. These lesions commonly involve the uterosacral ligaments, rectovaginal septum, and pelvic sidewalls [2]. Urinary tract endometriosis is uncommon, accounting for approximately 1-2% of all endometriosis cases. Among these, ureteral involvement represents nearly 10% [3]. Although rare, ureteral endometriosis carries a high risk of renal compromise due to progressive obstruction. The condition may be intrinsic, with direct infiltration of the ureteral muscularis or mucosa, or extrinsic, resulting from periureteral fibrosis and compression by adjacent deep infiltrating lesions. Extrinsic disease accounts for nearly 80% of reported cases [4]. A critical challenge in ureteral endometriosis is its silent progression. Gradual ureteral compression often produces minimal or nonspecific symptoms until advanced hydronephrosis develops. Studies have demonstrated that up to 30-50% of patients may present with significant renal impairment at diagnosis, and some are asymptomatic despite advanced obstruction [5]. Flank pain, pelvic pain, dysuria, and hematuria may occur but are inconsistent findings.

Preoperative identification remains difficult. Transvaginal ultrasonography with focused ureteral assessment may detect hydronephrosis but has limited sensitivity for early extrinsic disease. Magnetic resonance imaging provides improved detection of deep infiltrating lesions adjacent to the ureters and is currently considered the most sensitive noninvasive modality for mapping pelvic disease [6]. However, routine ureteral evaluation is not universally implemented in patients undergoing surgery for severe endometriosis. Management depends on the degree of obstruction. Ureterolysis may suffice in cases of mild extrinsic compression without intrinsic wall involvement. Advanced strictures require segmental resection with ureteroureterostomy or ureteroneocystostomy. Early intervention significantly improves renal preservation and long-term outcomes [7].

We report a case of distal ureteral endometriosis presenting three months after total abdominal hysterectomy, initially suspected to be iatrogenic ureteral injury. This case underscores the importance of structured preoperative ureteral assessment in patients with deep infiltrating endometriosis to prevent silent renal loss [8].

## Case presentation

A 42-year-old woman, gravida 5 para 2 abortus 3, presented with progressively worsening dysmenorrhea, chronic pelvic pain, deep dyspareunia, and heavy menstrual bleeding for five years. Over the preceding year, her pain had become continuous rather than cyclical. She reported passage of clots and fatigue secondary to anemia. There was no prior history of pelvic surgery, nephrolithiasis, or urinary tract disease. On examination, her body mass index was 24 kg/m². Abdominal examination revealed lower abdominal tenderness without guarding. Bimanual pelvic examination demonstrated a bulky, retroverted, and restricted uterus with bilateral uterosacral ligament nodularity and marked posterior fornix tenderness, suggestive of deep infiltrating disease. Laboratory evaluation revealed the following findings (Table 1).

**Table 1:** Baseline Laboratory Investigations The laboratory findings indicate iron deficiency anemia, characterized by low hemoglobin, microcytosis (low MCV), hypochromia (low MCH), and depleted iron stores (low serum ferritin), with normal renal function, consistent with anemia secondary to chronic blood loss or inadequate iron intake.

Parameter	Patient Value	Reference Range	Interpretation
Hemoglobin	9.2 g/dL	12–15 g/dL	Decreased
Mean Corpuscular Volume	72 fL	80–100 fL	Decreased
Mean Corpuscular Hemoglobin	24 pg	27–33 pg	Decreased
Serum Ferritin	10 ng/mL	15–150 ng/mL	Decreased
Serum Creatinine	0.8 mg/dL	0.6–1.1 mg/dL	Normal
Estimated Glomerular Filtration Rate	> 90 mL/minute/1.73 m^2^	> 90 mL/minute/1.73 m^2^	Normal
Blood Urea Nitrogen	12 mg/dL	7–20 mg/dL	Normal

Urinalysis showed no hematuria, no proteinuria, and no leukocyturia (0 to 5 white blood cells per high-power field).

**Figure 1:**
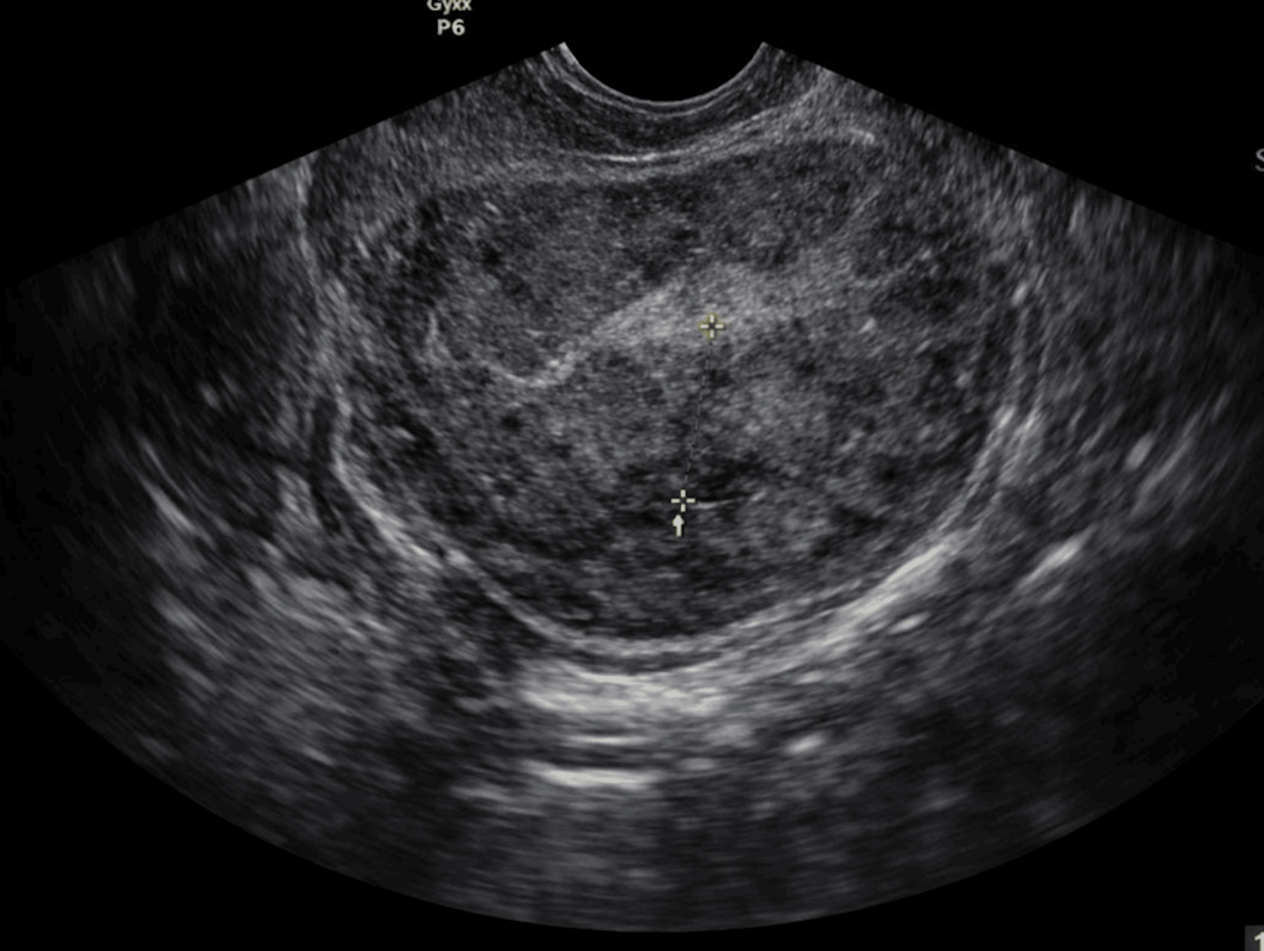
Preoperative Transvaginal Ultrasound The transvaginal ultrasound image demonstrates a markedly globular and bulky uterus that has lost its typical pear-shaped configuration, a finding highly characteristic of diffuse adenomyosis. Within the uterine wall, the myometrium exhibits a heteroechoic and mottled appearance, indicating an irregular internal texture rather than the smooth, uniform gray seen in a healthy organ. Subtle linear striations and fan-shaped shadowing are visible, representing the acoustic reflection of ectopic endometrial tissue and small cystic spaces embedded deep within the muscular layer. The junctional zone between the endometrial lining and the myometrium appears thickened and poorly defined, which further supports the diagnosis.

Transvaginal ultrasonography demonstrated a globular, bulky uterus with heteroechoic anterior and posterior myometrium. Endometrial thickness measured 6 mm. Small intramural fibroids were identified. A fibroid in the anterior myometrium of the lower uterine segment measured 8 mm. A posterior fundal fibroid measured 9.8 × 7.2 mm. Multiple nabothian cysts were noted in the cervix. Minimal fluid was seen in the endocervical canal, likely representing blood products. Bilateral adnexa were unremarkable. The radiologic impression was uterine adenomyosis with small intrauterine fibroids. No hydronephrosis was observed. Mild fibrotic thickening was noted adjacent to the expected course of the right distal ureter, but ureteral caliber appeared preserved. Dedicated ureteral phase imaging was not performed (Figure 1).

**Figure 2:**
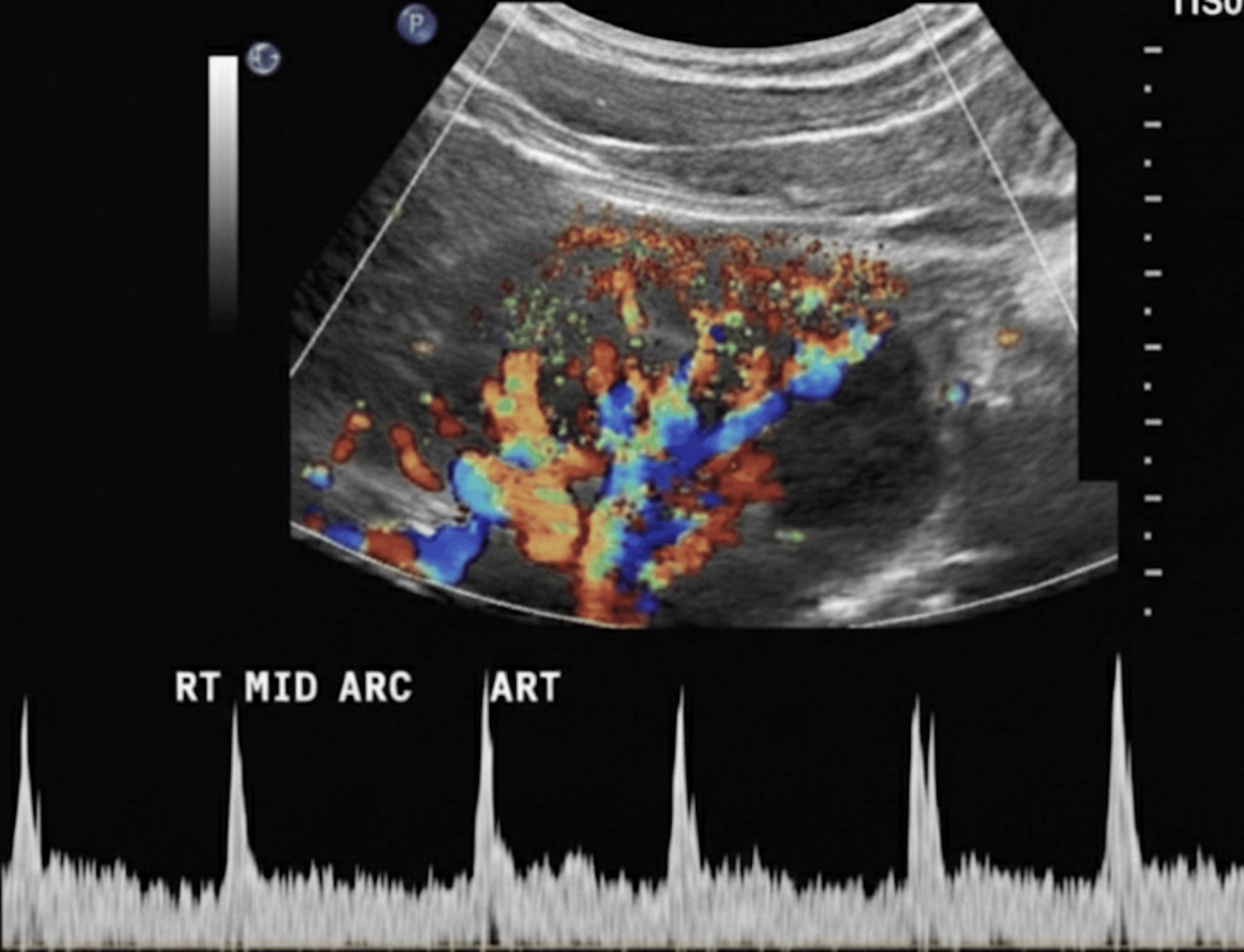
Preoperative Color and Spectral Doppler Ultrasound of the Right Kidney

As part of the preoperative evaluation for suspected renovascular hypertension, a renal Doppler ultrasound was performed before surgery. The right kidney measured 11.2 cm and the left kidney 11.0 cm. Both kidneys showed lobulated outlines with a few cortical scars but preserved corticomedullary differentiation. No backpressure changes or echogenic calculi were identified. On Doppler evaluation, bilateral main renal arteries demonstrated normal low-resistance waveforms with forward diastolic flow. Peak systolic velocity at the right renal artery hilum was 49 cm per second and 43.3 cm per second on the left. Acceleration times were 44 ms on the right and 66 ms on the left. Resistive indices were 0.62 on the right and 0.68 on the left. Intrarenal arterial waveforms were normal bilaterally. No suprarenal space-occupying lesion was seen. The impression was a normal renal Doppler study, with no evidence of renal artery stenosis or hydronephrosis (Figure 2).

After failure of medical therapy, including combined hormonal suppression and tranexamic acid, she opted for definitive surgical management. She underwent a total abdominal hysterectomy with bilateral salpingectomy. Intraoperative findings included dense adhesions between the uterus and rectosigmoid colon, thickened uterosacral ligaments, and fibrotic nodules extending to the right pelvic sidewall. The right ureter was identified at the pelvic brim and visually inspected. It appeared normal in diameter without obvious kinking or discoloration. No formal ureterolysis was performed. During the hysterectomy, there was no evidence of peritoneal endometriosis or spilt endometriosis. Also, the peritoneum was not disturbed. Estimated blood loss was 600 mL. The postoperative course was uneventful.

**Figure 3:**
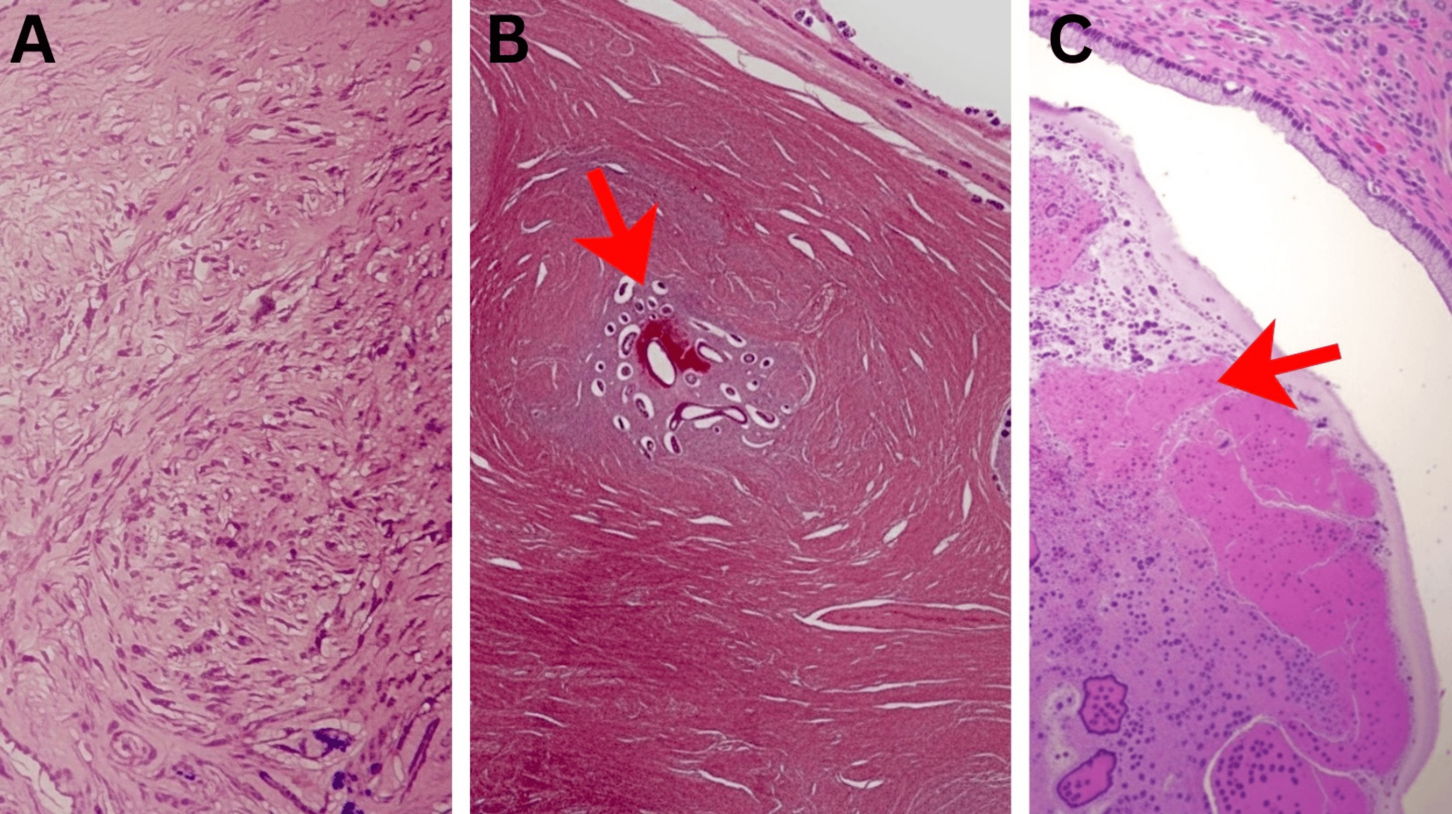
Microscopic Evaluations (A) Microscopic section of uterine tissue (leiomyoma) stained with Hematoxylin and Eosin (H&E). The image displays smooth muscle cells arranged in intersecting fascicles with prominent areas of eosinophilic, acellular hyaline degeneration I. Intramural and subserosal regions. Magnification: 400×. Scale bar = 100\µm. (B) Microscopic section of the uterine myometrium stained with H&E. The section reveals a focal nest of ectopic endometrial glands and associated stroma embedded within the hypertrophic smooth muscle fascicles (as indicated by the arrow), diagnostic of adenomyosis. Magnification: 100×. Scale bar = 200\µm. (C) Photomicrograph of cervical and paratubal tissue sections (H&E stain). The cervical section demonstrates chronic cervicitis with characteristic Nabothian cysts and areas of squamous metaplasia (as indicated by the arrow). The adnexal section confirms the presence of paratubal cysts lined by simple epithelium. Magnification: 100×. Scale bar = 500\µm.

Gross pathological examination revealed a hysterectomy specimen with bilateral fallopian tubes attached. The uterus measured 9 × 8 × 5.3 cm. The cut section showed multiple intramural and subserosal fibroids ranging from 0.3 to 1.0 cm in diameter. Endometrial thickness ranged from 0.2 to 0.4 cm. Myometrial thickness ranged from 0.5 to 2.6 cm. Nabothian cysts were identified in the cervix. One fallopian tube measured 4.0 cm and the other 5.5 cm in length. Paratubal cysts were identified bilaterally. Microscopic examination demonstrated leiomyomas with hyaline degeneration involving intramural and subserosal regions. The uterus showed adenomyosis with ectopic endometrial glands within the myometrium. Atrophic endometrium was present. Chronic cervicitis with nabothian cysts and focal squamous metaplasia was noted. Bilateral paratubal cysts were confirmed. No malignancy was identified (Figures 3A-C).

Three months postoperatively, the patient developed persistent right-sided flank pain radiating to the groin. The pain was dull, noncyclical, and progressively worsening. She denied fever, dysuria, or hematuria. Two empirical courses of oral antibiotics (nitrofurantoin 100 mg PO twice daily; trimethoprim-sulfamethoxazole (DS 160/800 mg)) were prescribed elsewhere for presumed urinary tract infection without symptomatic improvement. On evaluation, she had right costovertebral angle tenderness. Her laboratory investigations are shown in Table 2.

**Table 2:** Renal Function Tests at the Time of Postoperative Presentation

Parameter	Patient Value	Reference Range	Interpretation
Serum Creatinine	1.6 mg/dL	0.6–1.1 mg/dL	Elevated
Estimated Glomerular Filtration Rate	48 mL/minute/1.73 m^2^	> 90 mL/minute/1.73 m^2^	Decreased
Blood Urea Nitrogen	28 mg/dL	7–20 mg/dL	Elevated

Urinalysis showed 8 white blood cells per high-power field (0 to 5 per high-power field) without bacteriuria. Urine culture was sterile.

**Figure 4:**
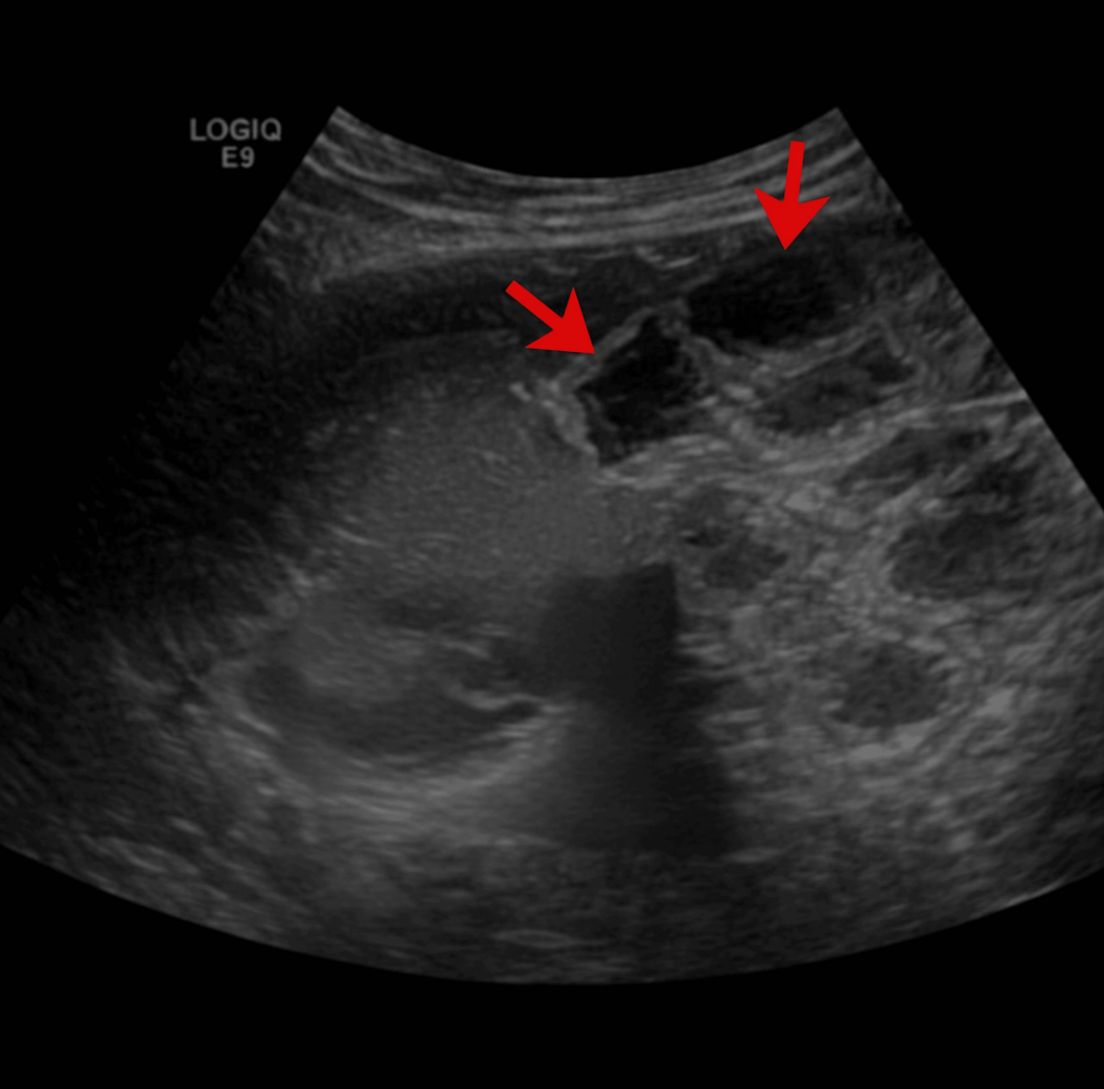
Ultrasound Abdomen This ultrasound image depicts severe hydronephrosis, characterized by the significant dilation of the renal pelvis and calyces (as indicated by the arrows), which appear as dark, fluid-filled (anechoic) pockets. The accumulation of trapped urine has distorted the kidney's normal architecture, creating a multiloculated appearance where the functional renal tissue, or parenchyma, appears thinned and compressed against the periphery.

Ultrasound abdomen demonstrated severe right hydroureteronephrosis with cortical thinning (Figure 4).

**Figure 5:**
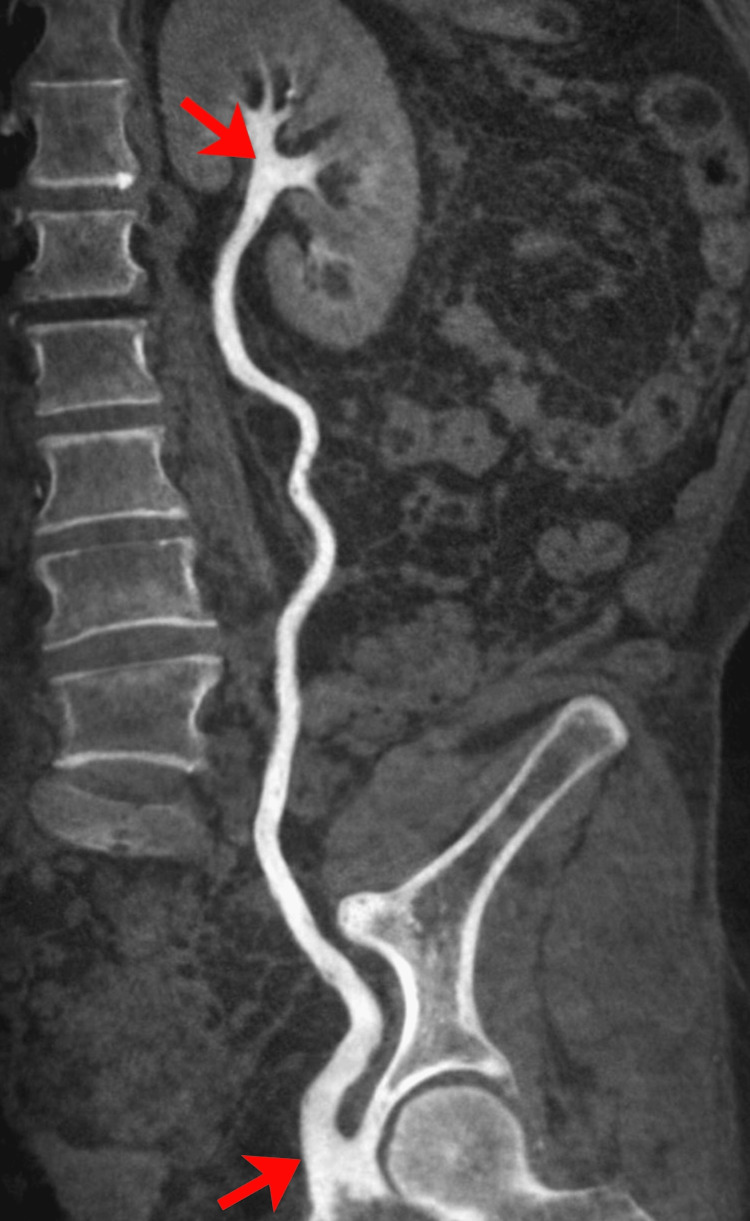
Contrast-Enhanced CT Urography Contrast-enhanced CT urography showing marked dilation of the right pelvicalyceal system (as indicated by the arrows). No calculus or surgical clip entrapment was identified.

Contrast-enhanced CT urography revealed marked dilation of the right pelvicalyceal system. No calculus or surgical clip entrapment was identified (Figure 5).

**Figure 6:**
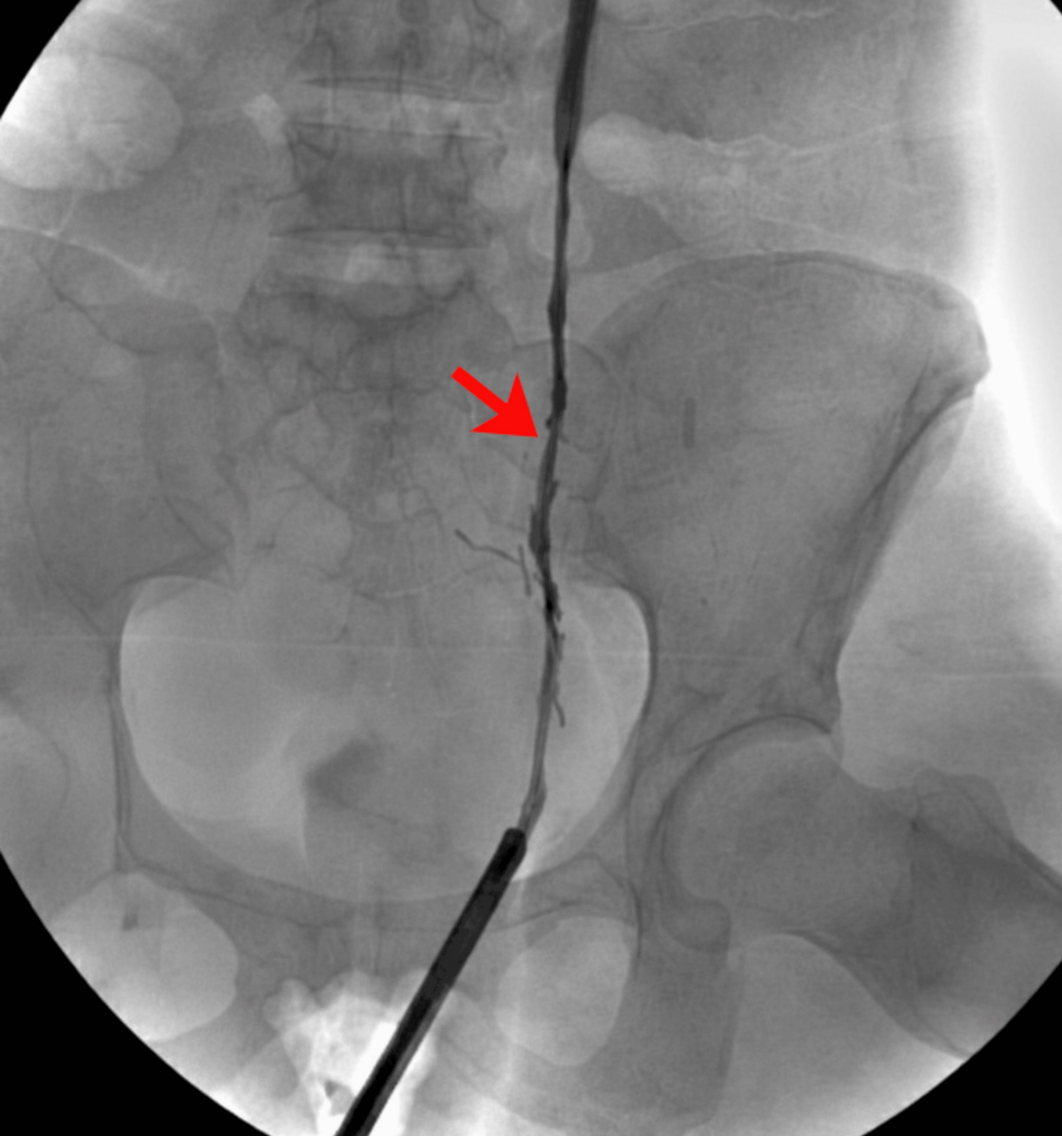
Retrograde Pyelography This retrograde pyelogram identifies a focal ureteric stricture in the mid-ureter (as indicated by the arrow), where the contrast column significantly narrows. The ureteric lumen measured approximately 8 mm proximal to the narrowed segment and was reduced to 2 mm at the level of the stricture, consistent with significant focal ureteric stenosis. The procedure involves injecting radiopaque dye upward from the bladder, which clearly delineates the transition from a patent lumen to this constricted segment against the lumbar vertebrae and ilium. There is an absence of any surgical clips/trauma.

Retrograde pyelography confirmed a high-grade ureteric stricture (Figure 6).

A right double-J stent was inserted, resulting in partial pain relief and a reduction of serum creatinine to 1.3 mg/dL over two weeks.

Definitive surgical management included right ureterolysis and segmental resection of a 2.5 cm fibrotic distal ureteral segment, followed by ureteroneocystostomy with psoas hitch. Intraoperative findings revealed dense circumferential fibrosis encasing the distal ureter without evidence of prior surgical transection or clip injury.

**Figure 7:**
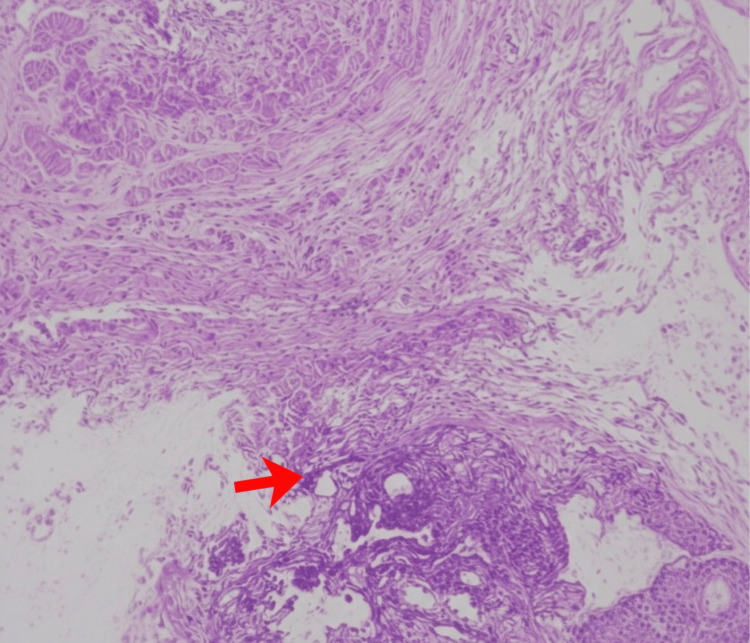
Histopathological Evaluation of the Excised Ureteral Segment Histopathological evaluation of the ureter stained with H&E, showing endometrial glands and stroma within the ureteral adventitia, accompanied by dense fibrosis and smooth muscle hyperplasia (as indicated by the arrow). No intrinsic mucosal invasion was observed. Magnification: 100×. Scale bar = 200\µm.

Histopathological evaluation of the excised ureteral segment demonstrated endometrial glands and stroma within the ureteral adventitia, accompanied by dense fibrosis and smooth muscle hyperplasia. No intrinsic mucosal invasion was observed (Figure 7).

These findings confirmed extrinsic ureteral endometriosis causing mechanical obstruction. Postoperatively, flank pain resolved. Serum creatinine improved to 1.0 mg/dL (reference range: 0.6-1.1 mg/dL), and follow-up imaging demonstrated resolution of hydronephrosis with preserved renal cortical thickness (Table 3).

**Table 3:** Clinical Timeline Table eGFR: Estimated Glomerular Filtration Rate

Clinical Phase	Serum Creatinine (mg/dL)	eGFR (mL/min/1.73 m²)	Interpretation
Preoperative Baseline	0.8	> 90	Normal renal function
Postoperative Presentation	1.6	48	Acute kidney injury secondary to obstruction
Post-reconstruction	1.0	> 90	Complete renal recovery

This chronological sequence confirms that renal imaging was normal preoperatively and supports the conclusion that previously unrecognized deep infiltrating ureteral endometriosis progressed postoperatively, mimicking iatrogenic ureteral injury.

## Discussion

Pathophysiological basis of ureteral involvement

Ureteral endometriosis represents one of the most severe manifestations of deep infiltrating endometriosis and carries a significant risk of silent renal compromise. Although urinary tract involvement accounts for approximately 1-2% of all endometriosis cases, ureteral disease constitutes nearly 10% of urinary tract endometriosis and is associated with progressive obstruction if not detected early [9]. Deep infiltrating endometriosis is defined as lesions penetrating more than 5 mm beneath the peritoneal surface and commonly involves the uterosacral ligaments, rectovaginal septum, and lateral pelvic sidewalls [10]. Because the distal ureter courses in close proximity to these structures, it is particularly vulnerable to secondary involvement.

Ureteral endometriosis is classified as intrinsic or extrinsic. Extrinsic disease accounts for nearly 80% of cases and results from periureteral fibrosis and compression rather than direct mucosal invasion [11]. Clement described that most ureteral lesions consist of dense fibrotic tissue surrounding the ureter, leading to progressive luminal narrowing [12]. Chronic, cyclical hemorrhage within ectopic endometrial implants induces sustained inflammatory activation, recruitment of macrophages, and secretion of profibrotic cytokines. This microenvironment stimulates fibroblast proliferation and excessive collagen deposition, resulting in concentric adventitial fibrosis and secondary muscular hypertrophy.

Pathological correlation in the present case

The hysterectomy specimen demonstrated adenomyosis characterized by ectopic endometrial glands and stroma embedded within hypertrophied myometrium, accompanied by smooth muscle hyperplasia. Multiple intramural and subserosal leiomyomas with areas of hyaline degeneration were also identified. Chronic cervicitis with nabothian cyst formation and focal squamous metaplasia was present. Although no direct ureteral involvement was identified in the uterine specimen, the presence of adenomyosis and fibrotic pelvic changes supports an estrogen-driven inflammatory milieu.

Definitive confirmation of ureteral endometriosis was obtained from histopathological evaluation of the resected distal ureter. Microscopic examination revealed endometrial glands lined by columnar epithelium, accompanied by endometrial-type stroma within the ureteral adventitia. Surrounding these ectopic elements was dense collagenous fibrosis with smooth muscle hyperplasia and focal chronic inflammatory infiltrates composed predominantly of lymphocytes. There was no evidence of mucosal ulceration or intrinsic epithelial invasion, confirming extrinsic disease. Hemosiderin-laden macrophages were identified in focal areas, indicating prior cyclical hemorrhage. The luminal narrowing was secondary to circumferential fibrotic thickening rather than an intraluminal mass effect. These findings align with previously described histomorphological patterns of extrinsic ureteral endometriosis [11,12].

Silent progression and renal compromise

A defining feature of ureteral endometriosis is its insidious progression. Donnez and colleagues emphasized that ureteral endometriosis may lead to “silent loss of renal function,” with substantial hydronephrosis present at diagnosis in a significant proportion of patients [13]. Because extrinsic compression develops gradually, symptoms may be mild or absent until obstruction becomes advanced. Published reports indicate that delayed diagnosis can result in cortical thinning and irreversible nephron loss [14].

In the present case, preoperative renal Doppler ultrasound demonstrated preserved renal size and architecture without hydronephrosis. The subsequent development of grade IV hydroureteronephrosis with elevated serum creatinine indicates progression of previously subclinical periureteral fibrosis or alteration in pelvic support structures following hysterectomy. Chronic obstruction causes increased intrapelvic pressure, reduced renal perfusion, tubular atrophy, and interstitial fibrosis. Early stenting and surgical reconstruction allowed improvement in renal function in this patient, highlighting the reversibility of obstruction when treated promptly.

Diagnostic considerations and imaging

Diagnosis of ureteral endometriosis remains challenging due to nonspecific symptoms and overlap with postoperative complications. In patients presenting with flank pain after hysterectomy, clinicians often prioritize iatrogenic ureteral injury. However, Darwish et al. reported that ureteral endometriosis may become clinically evident after pelvic surgery due to distortion of anatomy or acceleration of fibrotic processes [15]. Imaging findings in this case demonstrated abrupt distal tapering without evidence of surgical clip entrapment or transection. Intraoperative findings revealed dense fibrotic encasement rather than mechanical disruption.

Magnetic resonance imaging improves the detection of deep infiltrating lesions adjacent to the ureter and aids preoperative planning [16]. Chapron et al. emphasized that systematic mapping of posterior compartment disease increases identification of ureteral involvement and reduces unexpected postoperative complications [17]. The absence of dedicated ureteral sequences in preoperative imaging represents a contributory factor in delayed detection in this case.

Surgical management and outcome

Management strategies depend on the severity and anatomical extent of involvement. Mild extrinsic compression may respond to ureterolysis. However, high-grade strictures with functional impairment require segmental resection and ureteroneocystostomy. Zhang et al. demonstrated favorable renal preservation rates following timely surgical intervention [14]. In this patient, segmental resection with ureteroneocystostomy and psoas hitch achieved anatomical correction and functional recovery. Histopathological confirmation provided a definitive diagnosis and excluded iatrogenic injury.

Clinical implications

This case reinforces the importance of maintaining high clinical suspicion for ureteral involvement in patients with deep infiltrating endometriosis, particularly when uterosacral ligaments and pelvic sidewalls are affected. Even normal baseline renal imaging does not exclude early periureteral fibrosis. Structured preoperative assessment and multidisciplinary surgical planning may reduce delayed diagnosis and preserve renal function.

Limitations

The primary limitation of this case is the absence of dedicated preoperative ureteral-phase magnetic resonance imaging, which might have identified early periureteral fibrosis. Long-term renal functional follow-up beyond the early postoperative period is not yet available. However, the strength of this report lies in the clear chronological documentation of normal preoperative renal imaging, subsequent biochemical deterioration, radiologic confirmation of obstruction, intraoperative findings, and histopathological verification. This comprehensive correlation strengthens the causal interpretation and highlights the clinical relevance of early recognition in similar patients.

## Conclusions

Ureteral endometriosis is an uncommon but clinically significant manifestation of deep infiltrating endometriosis that may progress silently and culminate in substantial renal impairment if not recognized early. This case demonstrates how extrinsic periureteral fibrosis can evolve from subclinical narrowing with normal baseline renal imaging to advanced hydroureteronephrosis accompanied by rising serum creatinine and declining estimated glomerular filtration rate. Histopathological identification of endometrial glands and stroma within the ureteral adventitia, surrounded by dense concentric fibrosis without mucosal invasion, confirmed extrinsic ureteral endometriosis and excluded iatrogenic injury, an important distinction in the postoperative setting. The patient’s clinical course highlights the insidious nature of ureteral involvement, the limitations of routine imaging in detecting early periureteral disease, and the risk of attributing postoperative obstruction solely to surgical trauma. Prompt recognition, ureteral decompression, and definitive surgical reconstruction with ureteroneocystostomy enabled preservation and partial recovery of renal function, underscoring that timely intervention can prevent irreversible nephron loss.

While the urothelium’s natural resistance to invasion often favors conservative stenting, our case demonstrates that segmental resection remains a necessary intervention when high-grade obstruction or failure of prior measures is present. Given the chronic and recurrent nature of endometriosis, surgical decisions must balance radical excision for symptom relief with the preservation of long-term renal function through the most appropriate, patient-specific technique. This case reinforces the need for heightened suspicion, structured preoperative ureteral evaluation in patients with deep posterior compartment disease, and coordinated multidisciplinary management to minimize delayed diagnosis and long-term renal sequelae.
